# Molecular dynamics of pre-germinative metabolism in primed eggplant (*Solanum melongena* L.) seeds

**DOI:** 10.1038/s41438-020-0310-8

**Published:** 2020-06-01

**Authors:** Chiara Forti, Valentino Ottobrino, Laura Bassolino, Laura Toppino, Giuseppe Leonardo Rotino, Andrea Pagano, Anca Macovei, Alma Balestrazzi

**Affiliations:** 1 Department of Biology and Biotechnology ‘Lazzaro Spallanzani’, University of Pavia, Pavia, Italy; 2 CREA-Research Centre for Genomics and Bioinformatics, Montanaso Lombardo, LO Italy; 3 CREA-Research Centre for Cereal and Industrial Crops, Bologna, Italy; 4 Present Address: Institute of Plant Genetics, Polish Academy of Sciences, Poznan, Poland

**Keywords:** Plant physiology, DNA damage and repair

## Abstract

Seed priming, a pre-sowing technique that enhances the antioxidant/DNA repair activities during the pre-germinative metabolism, still retains empirical features. We explore for the first time the molecular dynamics of pre-germinative metabolism in primed eggplant (*Solanum melongena* L.) seeds in order to identify hallmarks (expression patterns of antioxidant/DNA repair genes combined with free radical profiles) useful to discriminate between high- and low-quality lots. The hydropriming protocol hereby developed anticipated (or even rescued) germination, when applied to lots with variable quality. ROS (reactive oxygen species) raised during hydropriming and dropped after dry-back. Upregulation of antioxidant/DNA repair genes was observed during hydropriming and the subsequent imbibition. Upregulation of *SmOGG1* (8-oxoguanine glycosylase/lyase) gene detected in primed seeds at 2 h of imbibition appeared as a promising hallmark. On the basis of these results, the investigation was restricted within the first 2 h of imbibition, to verify whether the molecular landscape was reproducible in different lots. A complex pattern of antioxidant/DNA repair gene expression emerged, reflecting the preponderance of seed lot-specific profiles. Only the low-quality eggplant seeds subjected to hydropriming showed enhanced ROS levels, both in the dry and imbibed state, and this might be a useful signature to discriminate among lots. The plasticity of eggplant pre-germinative metabolism stimulated by priming imposes a plethora of heterogeneous molecular responses that might delay the search for quality hallmarks. However, the information hereby gained could be translated to eggplant wild relatives to speed-up their use in breeding programs or other agronomical applications.

## Introduction

Seed priming is a low-cost, pre-sowing technique in which imbibition is carried out under controlled conditions in order to induce the antioxidant response and the DNA repair processes associated with the pre-germinative metabolism, but avoiding radicle protrusion and loss of desiccation tolerance^1–4^. Hydropriming and osmopriming (based on the use of osmotic solutions at low water potential that facilitate the control of water uptake) are widely used^4^. In the case of biopriming and chemopriming, the priming mixture is integrated with beneficial microorganisms or with conventional disinfectants and agrichemicals, respectively^4^. At the end of the treatment, primed seeds undergo dehydration (dry-back) and finally they are stored and/or commercialized. Enhanced crop yields resulting from primed seeds are generally due to increased tolerance to biotic and abiotic stresses that improves population density and individual plant performance. Owing to these benefits, the priming technology is gaining relevance as a strategy to address the current and future issues of sustainable crop production on degraded soils^5–7^. Despite the wide use of these treatments to improve seed quality, priming is still an empirical procedure. Such an undesirable feature delays the work of seed technologists, breeders, and seed bank operators. A deeper understanding of the molecular processes that control the seed response to priming will help the search for molecular hallmarks (namely genes, proteins, and metabolites) useful for predicting the effectiveness of novel pre-sowing treatments^8,9^. It is generally acknowledged that enhanced seed vigor depends on a robust antioxidant response^10,11^ whereas effective DNA repair contributes to genome maintenance, another key determinant of seed quality^12,13^. During seed imbibition, there is increased oxidative DNA damage, namely enhanced levels of 7,8-dihydro-8-oxoguanine (8-oxodG), as reported in *Medicago truncatula*^9^ and the upregulation of genes belonging to the BER (base excision repair) pathway as *OGG1* (8-oxoguanine glycosylase/lyase), and *FPG* (formamidopyrimidine-DNA glycosylase)^14^. OGG1 (EC: 3.2.2-4.2.99.18) is a bifunctional DNA glycosylase/lyase, typically found in eukaryotes that catalyzes the release of 8-oxo-dG and the cleavage of DNA at the resulting abasic site. The role of OGG1 in counteracting oxidative DNA damage accumulated during seed desiccation has been documented^15,16^. FPG (EC: 3.2.2.23) is responsible for the excision of 8-oxo-dG and FAPy (2,6-diamino-4-hydroxy-5-formamidopyrimidine) lesions. Another DNA repair gene, linked to BER, encodes tyrosyl-DNA phosphodiesterase 1 (TDP1; EC: 3.1.4) that removes the stalled topoisomerase I-DNA by cleaving a 3′-phosphotyrosyl bond. In animal cells, the TDP1 enzyme also interacts with BER proteins for the repair of oxidized DNA lesions^17^. According to the literature, the plant *TDP1α* gene, coding for the highly conserved TDP1α isoform, might be involved in the seed response to priming as it is upregulated during seed imbibition in presence/absence of oxidative stress^9,18–20^. These studies show that the BER pathway is a source of hallmarks useful to improve priming treatments.

Eggplant (*Solanum melongena* L.) is worldwide cultivated and appreciated for its highly valuable nutritional components that contribute to disease prevention^21,22^. *Solanum* species are mainly propagated by seed. However, dormancy and low seed germination rate/uniformity are found in *S. melongena* accessions and their wild relatives^23–27^, and these are undesired features that affect the use of wild relatives as rootstocks for eggplant and other compatible crop species^28,29^. These issues severely impact breeding programs, with a huge economic impact on seed industries that need to increase product performance while optimizing the use of economic resources.

In this context, seed priming is used to enhance germination and provide faster emergence, uniform stands, and stress tolerant plants^4^. Several reports are currently available, describing the effects of different priming protocols on eggplant seed germination and seedling emergence^30–35^, however the molecular characterization of the seed response to priming is still lacking. In the present work, we investigated these issues using the *S. melongena* inbred line ‘67/3’, for which both the reference genome and transcriptome are available^36^.

In this study, the role of BER/antioxidant genes as tools to monitor seed priming, and its impact on germination was assessed in the ‘67/3’ line by comparing the response of seed lots of different quality. Furthermore, the dynamics of ROS accumulation in primed versus unprimed seeds were evaluated as a possible quality hallmark.

## Results

### Hydropriming promotes germination of ‘67/3’ seeds

**Fig. 1:**
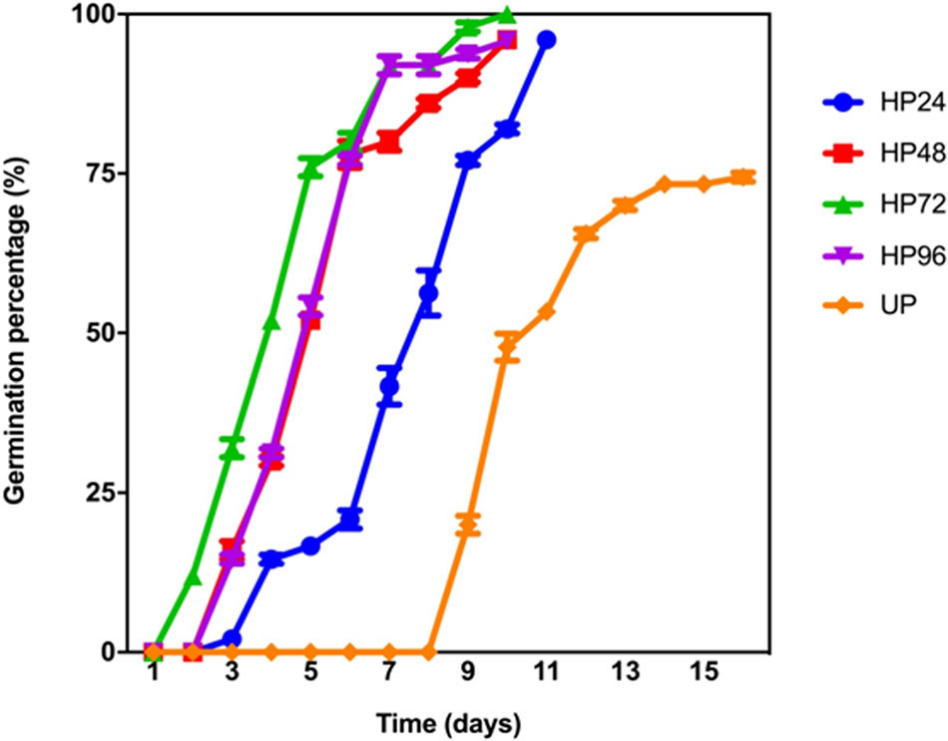
Hydropriming accelerates germination of ‘67/3’ seeds. For priming, seeds were soaked in H_2_O for 24, 48, 72, and 96 h under aeration and subsequently re-dried (dry-back) until the weight of the dry seed was reached. Unprimed (UP) and primed (HP) dry seeds were then immediately used for germination tests. Values are expressed as mean ± SD of three independent replications with 20 seeds for each replication. Statistical analysis was carried out using two-way ANOVA (*F* = 45.66; DF = 4; ***P = 0.0004). Comparison between UP and HP24, HP48, HP72, and HP96 were carried out using the post-hoc Tukey’s HSD test (*P* ≤ 0.05) (see Supplementary Information, Supplementary Table S11)

Hydropriming (HP) was first tested on ‘67/3’ seeds collected in 2014, hereby named Seed Lot 1 (SL1). Seeds soaked in H_2_O for 24, 48, 72, and 96 h (treatments HP24, HP48, HP72, and HP96) were subjected to dry-back (DB, 2 h) and then immediately used for germination tests. As shown in Fig. 1, unprimed (UP) seeds started germination at 9 days from the beginning of imbibition and reached the maximum germination percentage (74.44 ± 0.71%) at 16 days. Hydropriming always resulted in anticipated germination compared to UP seeds. In the case of primed seeds, germination occurred at 5 days (40.00 ± 0.00%, HP24), 4 days (6.00 ± 0.71%, HP48), 3 days (2.00 ± 0.71%, HP72), and 2 days (4.00 ± 0.00%, HP96) from the beginning of imbibition.

A more detailed analysis of the germination profiles was performed as described by Ranal and Garcia de Santana^37^ (Table 1). *G* (Germinability) reached 100% for HP24, HP48, and HP72, whereas a slight reduction (98.00 ± 2.80%) was observed for HP96. Overall, HP72 was the best treatment, based on MGT (mean germination time) (4.48 ± 0.00 days), and CVG (coefficient of variation of velocity of germination) (468.00 ± 0.15%) parameters. Both the lowest MGR (mean germination rate) and *U* (uncertainty) values, recorded for HP72 (2.14 × 10^−3^ ± 0.00 day^−1^ and 1.22 ± 0.20 bit, respectively) were indicative of synchronized germination (Table 1). *U* is defined as the uncertainty associated to the distribution of the relative frequency of germination or the degree of spreading of germination through time. It is calculated based on the formula reported in Supplementary Table S6, where *f*_*i*_ is the relative frequency of germination, *n*_*i*_ the number of seeds germinated on the day *i*, and *k* the last day of observation. The unit is bit that is a binary measurement that covers germinated/non germinated seeds. *Z* reflects the synchrony of one seed with another included in the same replication of one treatment or the degree of germination overlapping. *Z* = 1 when the germination of all seeds occur at the same time and *Z* = 0 when at least two seeds could germinate, one at each time. It is calculated based on the formula reported in Supplementary Table S6.

**Table 1:** Germination parameters calculated based on results of germination tests carried out on ‘67/3’ seeds treated with hydropriming (HP) for increasing time (24, 48, 72, and 96 h) and unprimed (UP) seeds

Treatment	*G* (%)	MGT (days)	CVG (%)	MGR (day^−1^)	*U* (bit)	*Z* (unit less)
HP 24 h	100.0 ± 0.00*	6.56 ± 0.05**	288.00 ± 3.41*	3.4 ×10^−3^ ± 7.07 ×10^−5^*	2.08 ± 0.00	0.26 ± 0.00
HP 48 h	100.0 ± 0.00*	5.78 ± 0.08**	339.76 ± 6.29**	2.9 ×10^−3^ ± 5.65 ×10^−5^*	1.70 ± 0.38	0.34 ± 0.10
HP 72 h	100.0 ± 0.00*	4.48 ± 0.00***	468.00 ± 0.15*	2.1 ×10^−3^ ± 0.00*	1.22 ± 0.20	0.31 ± 0.30
HP 96 h	98.0 ± 2.80*	4.77 ± 0.18*	426.02 ± 33.09	2.3 ×10^−3^ ± 1.98 ×10^−4^**	2.01 ± 0.38	0.25 ± 0.05
UP	74.4 ± 1.57	10.58 ± 0.01	220.38 ± 6.55	4.5 ×10^−3^ ± 1.41 ×10^−4^	2.22 ± 0.02	0.23 ± 0.01

Asterisks indicate statistically significant differences determined using Student’s *t*-test (*P < 0.05; **P < 0.01, ***P < 0.001)

*G* germinability, *MGT* mean germination time, *CVG* coefficient of velocity of germination, *MGR* mean germination rate, *U* uncertainty, *Z* synchronization index, h hours

Biometric parameters measured on 7-day-old ‘67/3’ seedlings developed from HP72 seeds highlighted significant changes only in seedling fresh weight (Supplementary Table S1). Based on this results, the HP72 treatment was selected for further investigations.

### Hydropriming rescues germination in low-quality ‘67/3’ seed lots

The HP72 protocol was applied to ‘67/3’ seed lots collected during the subsequent years (2015, 2016, 2017, 2018, named SL2, SL3, SL4, and SL5, respectively). Germination tests were carried out and the resulting data were used to calculate the specific germination parameters. As shown in Table 2, in the case of SL2, UP seeds were not able to germinate, whereas the HP72 treatment rescued germination. The recorded values for the HP72 sample were 55.56 ± 21.42% (*G*), 6.52 ± 0.86 days (MGT), 59 ± 42.48% (CVG), 0.0269 ± 0.022 day^−1^ (MGR), 1.61 ± 0.87 bit (*U*), and 0.34 ± 0.28 (*Z*). As for SL3, a significantly lower MGT (6.20 ± 0.00 days) was observed for the primed seeds compared to UP (8.95 ± 0.91 days). Except for MGR, all the other recorded traits in HP72 seeds were significantly different from UP seeds whereas only slight differences were noticed in *U* values (UP, 2.39 ± 0.05 and HP72, 2.04 ± 0.16 bit, respectively) (Table 2). SL4 showed a late germination profile, however the HP72 treatment could anticipate germination as evidenced by MGT values (UP, 28.56 ± 1.34 days; HP72, 25.51 ± 0.79 days), whereas no effects were evidenced in other traits, including synchronization (recorded *U* values: UP, 2.53 ± 0.14 bit; HP72, 2.74 ± 0.18 bit). As for SL5, germination was anticipated (MGT values of 11.05 ± 1.10 and 8.84 ± 0.87 days, for UP and HP72 samples, respectively), although no significant effects on synchronization were detected. By considering the overall data on germination profiles, the ‘67/3’ seeds were grouped as ‘high-quality’ (SL1, SL3, SL5) and low-quality (SL2, SL4) lots.

**Table 2:** Germination parameters calculated for different ‘67/3’ seed lots collected during 2015 (SL2), 2016 (SL3), 2017 (SL4), 2018 (SL5) and treated with hydropriming (HP72 protocol)

Seed lot	*G* (%)		MGT (days)		CVG (%)		MGR (day^−1^)		*U* (bit)		*Z* (unit less)	
	UP	HP72	UP	HP72	UP	HP72	UP	HP	UP	HP72	UP	HP72
SL2	0	55.56 ± 21.42	0	6.52 ± 0.86	0	59.00 ± 42.48	0	0.030 ± 0.02	0	1.61 ± 0.87	0	0.340 ± 0.28
SL3	100.00 ± 0.00	100.00 ± 0.00	8.95 ± 0.91	6.20 ± 0.00*	76.34 ± 17.85	148.70 ± 0.94*	0.005 ± 0.003	0.007 ± 0.00	2.39 ± 0.05	2.04 ± 0.16*	0.18 ± 0.00	0.230 ± 0.01*
SL4	77.78 ± 19.24	74.13 ± 11.43	28.56 ± 1.34	25.51 ± 0.79*	62.70 ± 12.00	65.00 ± 10.59	0.005 ± 0.003	0.016 ± 0.00	2.53 ± 0.14	2.74 ± 0.18	0.19 ± 0.02	0.077 ± 0.04
SL5	97.78 ± 3.85	100.00 ± 0.00	11.05 ± 1.10	8.84 ± 0.87	42.03 ± 9.86	77.61 ± 17.23*	0.025 ± 0.006	0.013 ± 0.00	2.50 ± 0.29	2.57 ± 0.11	0.15 ± 0.05	0.136 ± 0.005

Asterisks indicate statistically significant differences determined using Student’s *t*-test (*P < 0.05)

*G* germinability, *MGT* mean germination time, *CVG* coefficient of velocity of germination, *MGR* mean germination rate, *U* uncertainty, *Z* synchronization index, UP unprimed seeds, SL seed lot

### Hydropriming boosts ROS accumulation

**Fig. 2:**
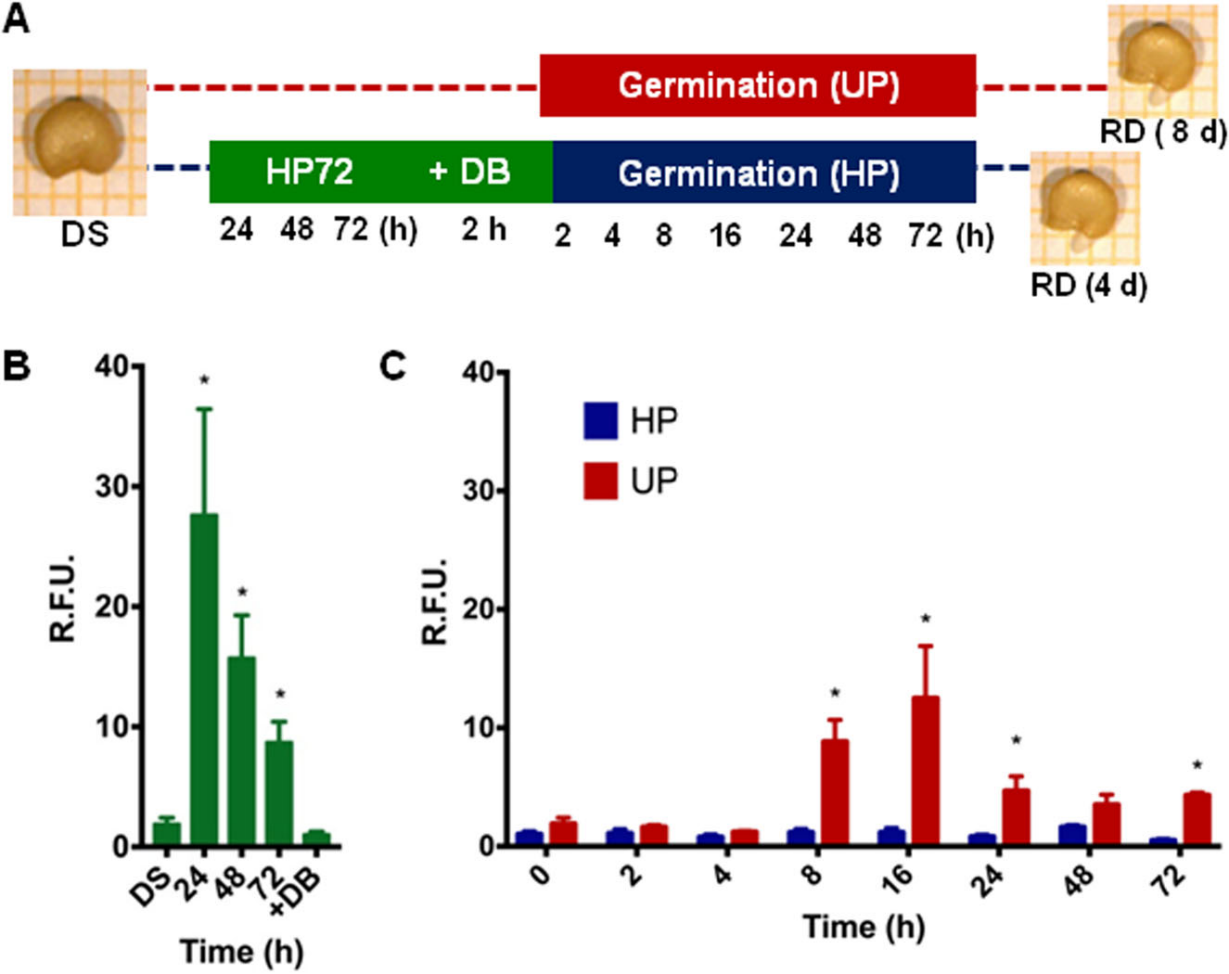
Impact of hydropriming on ROS levels in ‘67/3’ seeds. **a** Experimental design showing the selected timepoints for molecular analyses. Seeds were collected during priming (HP72 protocol) at 24, 48, and 72 h, as well as at the end of dry-back (DB, 2 h). Germination of unprimed (UP) and primed (HP) seeds was monitored by collecting samples throughout imbibition at 2, 4, 8, 16, 24, 48, and 72 h. The last timepoint, corresponding to the phenological stage of radicle protrusion (RD), was anticipated at 4 days for the primed seeds, compared to unprimed (8 days). ROS levels were measured during hydropriming **b** and subsequent germination **c** at the selected timepoints using the DCFH-DA fluorescent dye. Values are expressed as mean ± SD of three independent replications with 20 seeds for each replication. Asterisks indicate statistically significant differences determined using Student’s *t*-test (*P* < 0.05). DS dry seed, R.F.U. relative fluorescence unit, ROS reactive oxygen species, DCFH-DA dye 2′,7′-dichlorofluorescein diacetate

The experimental design set up for the molecular analyses is shown in Fig. 2a. Samples were collected at 24, 48, and 72 h during HP and subsequently after the dry-back (DB) step. During germination tests, UP and HP seeds were collected at 2, 4, 8, 16, 24, 48, and 72 h from the beginning of imbibition and at the radicle protrusion stage (RD) (Fig. 2a). On the basis of the germination parameters, the sampling procedure for the radicle protrusion stage was conducted earlier in HP seeds (at 4 days from the beginning of imbibition) than for UP seeds (at 8 days from the beginning of imbibition) (Fig. 2a).

ROS (reactive oxygen species) levels were measured using the DCFH-DA fluorescent dye in dry and imbibed ‘67/3’ seeds at selected timepoints. These experiments were performed using SL5, both dry seeds (DS) and seeds collected throughout the HP treatment (24, 48, 72 h) (Fig. 2b). HP triggered a significant (*P* = 0.04) ROS accumulation at 24 h (27.63 ± 8.82 relative fluorescence unit (R.F.U.)), 14.62-fold compared to DS (1.89 ± 0.53 R.F.U.). At 48 and 72 h, the recorded ROS levels were still significantly higher (15.72 ± 3.55 and 8.71 ± 1.70 R.F.U., respectively; *P* = 0.02 and 0.01) (Fig. 2b). Following dry-back, ROS levels further decreased compared to the 72 h timepoint, reaching an estimated value of 1.02 ± 0.25 R.F.U. (*P* = 0.08), similar to those detected in the dry seeds (Fig. 1c, +DB). A different pattern was detected in the UP samples since a peak in ROS levels was observed at 8 and 16 h of imbibition (8.84 ± 1.83 and 12.50 ± 4.40 R.F.U., respectively; *P* = 0.02 and 0.04), compared to the DS (1.89 ± 0.53 R.F.U.) (Fig. 2c, UP). ROS levels were decreased at the subsequent timepoints, from 24 to 72 h, although they appeared still significantly higher (*P* = 0.03 and 0.00008), compared to dry seeds (4.68 ± 1.23 R.F.U., 24 h; 3.50 ± 0.86 R.F.U., 48 h; 4.31 ± 0.23 R.F.U., 72 h) (Fig. 2c, UP). The RD stage marks the end of germination and features extensive metabolic changes other than those associated with the pre-germinative metabolism^20^. For this reason, this stage was not considered for ROS measurements. On the basis of the reported data, the HP72 treatment featured enhanced ROS accumulation within the first 24 h, whereas no consistent changes in ROS pattern were associated with the accelerated germination profile of HP seeds.

### Hydropriming induces temporally distinct waves of upregulation of antioxidant and BER genes

**Fig. 3:**
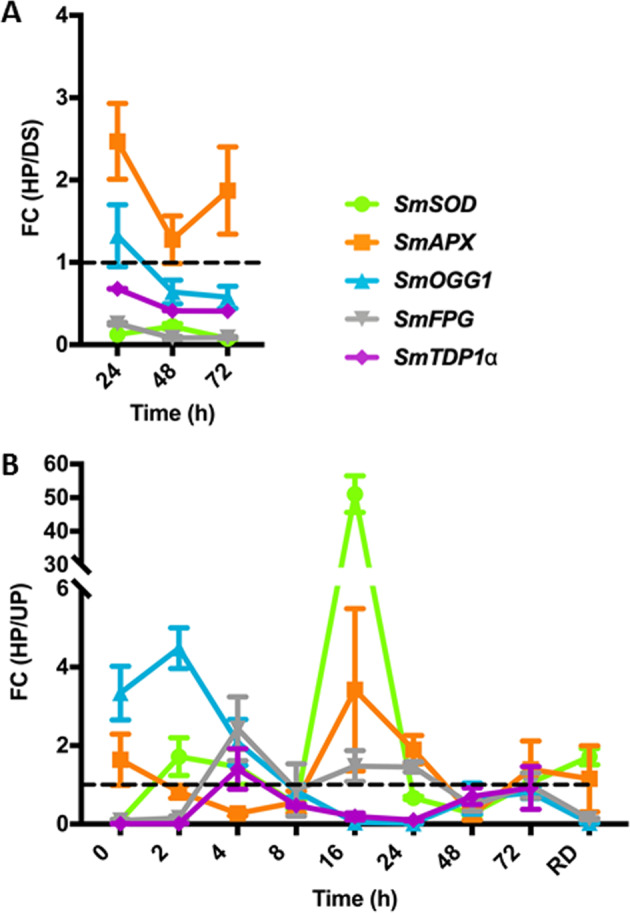
Impact of hydropriming on the expression profiles of antioxidant and DNA repair genes in ‘67/3’ seeds. **a** Results of qRT-PCR analyses performed on seeds collected during the HP72 treatment (at 24, 48, and 72 h) and compared to the DS are shown as expression changes of antioxidant and BER genes. Values represent fold changes of transcript levels where FC = primed seed (HP)/dry seed (DS) and FC(HP/DS) values <1 or >1 correspond to gene downregulation or upregulation, respectively (dashed line). Relative gene expression values are shown in Supplementary Table S5. **b** Results of qRT-PCR analyses performed on HP and UP seeds collected during the germination test (0, 2, 4, 8, 16, 24, 48, and 72 h) and at the radicle protrusion stage (RD) are shown as expression changes of antioxidant and BER genes. Values represent fold changes of transcript levels where FC = primed seed (HP)/unprimed seeds (UP) and FC(HP/UP) values <1 or >1 correspond to gene downregulation or upregulation, respectively (dashed line). Relative gene expression values are shown in Supplementary Table S6. Values are expressed as mean ± SD of three independent replications with 20 seeds for each replication. Results of the Student’s *t*-test highlighting statistically significant differences are shown in Supplementary Table S4. Sm *Solanum melongena*, SOD superoxide dismutase, APX ascorbate peroxidase, OGG1 8-oxoguanine glycosylase/lyase, FPG formamidopyrimidine-DNA glycosylase, TDP1α tyrosyl-DNA phosphodiesterase 1α

In order to evaluate the effects of the HP72 treatment on the seed antioxidant defense and DNA repair response, the expression profiles of selected genes, known to be involved in the seed pre-germinative metabolism^9,19,20^ were investigated using qRT-PCR. Putative orthologs corresponding to *S. melongena* (*Sm*) *SmSOD* (*superoxide dismutase)*, *SmAPX* (*ascorbate peroxidase*), *SmOGG1* (*8-oxoguanine glycosylase/lyase*), *SmFPG* (*formamidopyrimidine-DNA-glycosylase*), and *SmTDP1α* (*tyrosyl-DNA phosphodiesterase*) were identified as best-hits for *A. thaliana* and *M. truncatula* proteins in the Eggplant Genome Browser^36^. Results of qRT-PCR analyses performed on ‘67/3’ samples (harvested in 2018) collected during the HP72 treatment, and compared to the DS, are shown in Fig. 3a as expression changes of antioxidant and BER genes. Values represent fold changes of transcript levels where FC = primed seed (HP)/dry seed (DS). FC(HP/DS) values <1 or >1 correspond to gene downregulation or upregulation, respectively (Fig. 3a, dashed line). Relative gene expression values are shown in Supplementary Table S2. When considering the effects of HP72 treatment along the tested timepoints, compared to DS, the *SmSOD* gene was downregulated, as indicated by the observed FC(HP/DS) values (<1), whereas upregulation of the *SmAPX* gene was evident after 24 h of treatment. Among the tested BER genes, only *SmOGG1* showed early upregulation at 24 h (Fig. 3a).

Results of qRT-PCR analyses performed on the ‘67/3’ samples, both HP and UP seeds, collected during the germination test are shown in Fig. 3b, as expression changes of antioxidant and BER genes. Values represent fold changes of transcript levels where FC = primed seed (HP)/unprimed seeds (UP). FC(HP/UP) values <1 or >1 correspond to gene downregulation or upregulation, respectively (Fig. 3b, dashed line). Gene expression relative values are shown in Supplementary Table S3. When comparing HP seeds subjected to dry-back with UP dry seeds (Fig. 3b, 0 h), the observed FC(HP/UP) values indicated downregulation of the *SmSOD* gene as well as the HP-dependent upregulation of *SmAPX* and *SmOGG1* genes. The FC(HP/UP) value of *SmOGG1* significantly increased following dry-back (from 0.80 ± 0.15 at 72 h up to 3.34 ± 0.70), suggesting for enhanced expression during the dehydration step. Besides the early upregulation of *SmAPX* and *SmOGG1* genes, the impact of the HP treatment on seed imbibition is evidenced by distinct waves of responses. At 2 h, the *SmOGG1* gene was still upregulated in HP seeds whereas the recorded increase in FC(HP/UP) for the *SmSOD* gene (1.72 ± 0.48), compared to 0 h, highlights the influence of the treatment on this antioxidant component of the eggplant pre-germinative metabolism (Fig. 3b, 2 h). At 4 h, the treatment resulted into a distinctive response of BER genes. Indeed, despite the observed decrease in the FC(HP/UP) value, the *SmOGG1* gene was still upregulated at this timepoint. HP72 triggered also the expression of *SmFPG* and *SmTDP1α* genes, based on the estimated FC(HP/UP) values of 2.43 ± 0.81 and 1.40 ± 0.52, respectively (Fig. 3b, 4 h). At 16 h, a new wave of response was observed featuring upregulation of the antioxidant gene *SmAPX* with an estimated FC(HP/UP) value of 3.42 ± 2.07 as well as a very strong response of *SmSOD* gene (FC(HP/UP) = 51.07 ± 5.43) (Fig. 3b, 16 h). The slight fluctuations detected at the end of the experiment, at 48 and 72 h, and the changes observed at the radicle protrusion stage (RD) showed that the impact of HP was apparently diminished when approaching the end of germination. To better clarify the dynamics of the seed response resulting from the concomitant changes in the *SmOGG1* mRNA and ROS levels, the ratio between the *SmOGG1* transcript level and the ROS amount (OGG1:ROS) was calculated. A significant increase in the OGG1:ROS ratio occurred in HP seeds, compared to UP, during early imbibition (from 0 to 4 h) (Supplementary Fig. S1, HP). The observed differences in gene expression and ROS patterns suggest that the temporal ‘window’ spanning the first hours of imbibition provides information useful to predict the efficacy of hydropriming. To further explore the potential of this ‘window’, ROS profiles and gene expression analyses were carried in the ‘67/3’ seed lots previously characterized, showing variable quality.

**Fig. 4:**
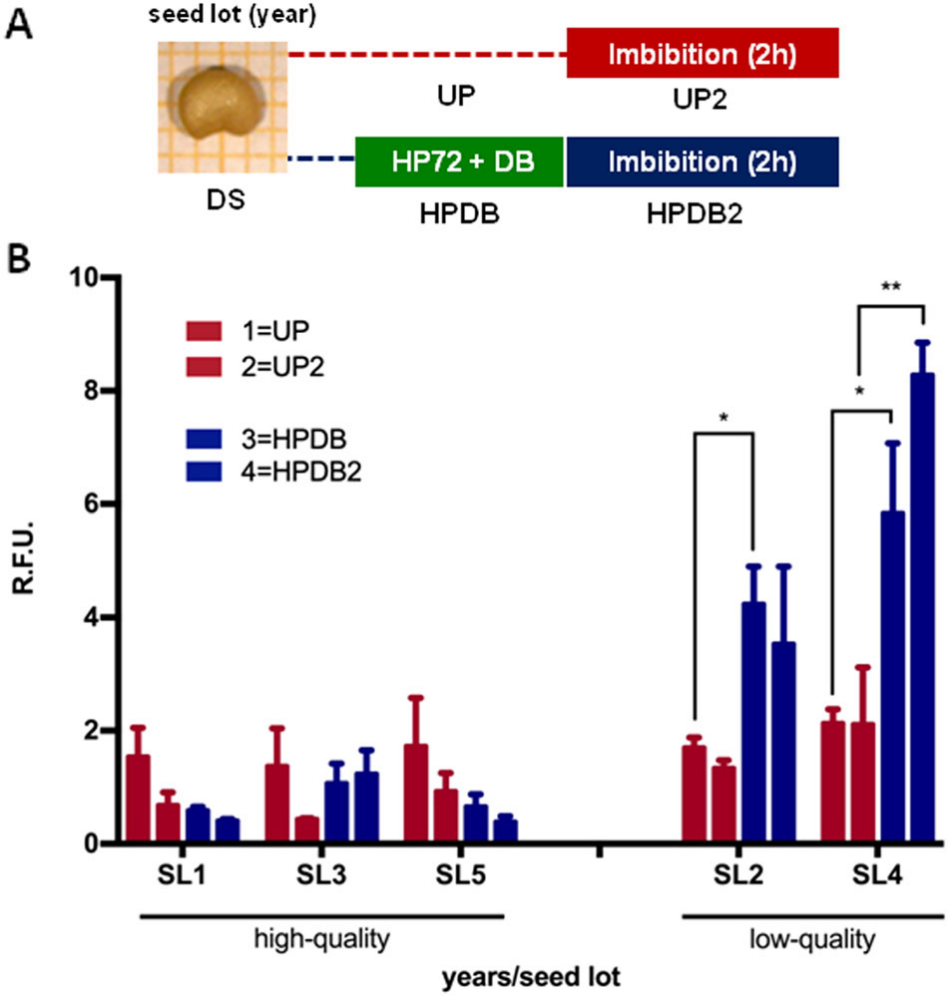
ROS levels were assessed in the different ‘67/3’ seed lots previously characterized for their germination profiles (high-quality and low-quality lots) and response to hydropriming. **a** The experimental design is shown: for each lot, the unprimed samples included dry seeds (UP) as well as rehydrated seeds collected at 2 h of imbibition (UP2), whereas the primed samples included seeds that underwent the HP72 treatment followed by dry-back (HPDB) and seeds collected at 2 h of imbibition (HPDB2). **b** ROS levels were measured at the selected timepoints using the DCFH-DA fluorescent dye. Values are expressed as mean ± SD of three independent replications with 20 seeds for each replication. Asterisks indicate statistically significant differences determined using Student’s *t*-test (*P* < 0.05). DS, dry seed. R.F.U. relative fluorescence unit, ROS reactive oxygen species, DCFH-DA dye 2′,7′-dichlorofluorescein diacetate, SL seed lot

### Correlation between seed quality and ROS profiles

ROS levels were assessed in high- and low-quality ‘67/3’ seed lots. The experimental design is shown in Fig. 4a. For each lot, the unprimed samples included dry seeds (UP) and rehydrated seeds collected at 2 h of imbibition (UP2), whereas the primed samples included seeds that underwent the HP72 treatment followed by dry-back (HPDB) and seeds collected at 2 h of imbibition (HPDB2). The sampling was limited to this early timepoint with the aim to speed-up the prediction of the seed response and reduce costs, in view of possible future applications. Comparable ROS levels were detected in the UP seeds of high-quality’ lots (1.54 ± 0.51 R.F.U., SL1; 1.37 ± 0.66 R.F.U., SL3; 1.73 ± 0.85 R.F.U., SL5). At 2 h of imbibition (UP2) ROS levels were decreased in SL1 and SL3 (0.68 ± 0.23 and 0.44 ± 0.01 R.F.U., respectively) compared to dry seeds. A similar trend was observed also in SL5 (Fig. 4b). The impact of HP72 treatment on the high-quality seed lots was not apparently relevant as ROS levels detected in both HPDB and HPDB2 samples did not exceed the amount measured in the UP seeds whereas the low-quality seed lots showed a significant (*P* = 0.02, 2015 and *P* = 0.03, 2017) enhancement following the HP72 treatment (Fig. 4b). SL2 and SL4 did not show high germinability. In the case of SL2, no germination at all was observed while the HP72 treatment induced germination, although at a low percentage (55.56 ± 21.42%) (Table 2). In the other case (SL4), the seed lot showed suboptimal germination (77.78 ± 19.24%) and there was no benefit from hydropriming (Table 2). It is possible that the poor-quality germination performance of SL2 and SL4 correlates with high ROS levels detected in the seeds subjected to hydropriming. On the other hand, the low ROS profiles in SL1, SL3, and SL5 should reflect their high-quality germination performance.

### Impact of hydropriming on the expression of antioxidant/BER genes in high- and low-quality eggplant seed lots

**Fig. 5:**
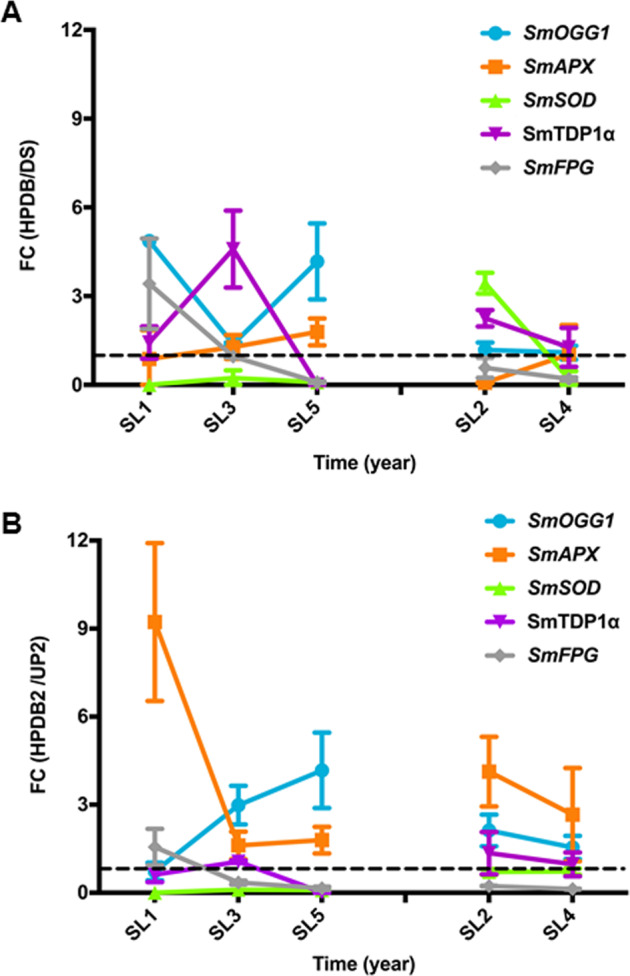
Impact of hydropriming on the expression profiles of antioxidant and DNA repair genes in different ‘67/3’ seed lots. **a** Results of qRT-PCR analyses performed on seeds collected according to the experimental design shown in Fig. 4a are reported as expression changes of antioxidant and BER genes. Values represent fold changes of transcript levels where FC = primed and dried-back seed (HPDB)/dry seed (DS) and FC(HPDB/DS) values <1 or >1 correspond to gene downregulation or upregulation, respectively (dashed line). Relative gene expression values are reported in Supplementary Table S5. **b** Results of the qRT-PCR analysis performed on imbibed seeds are shown as expression changes of antioxidant and BER genes. Values represent fold changes of transcript levels where FC = primed seed (HP_2h_)/unprimed seed (UP_2h_) and FC(HP_2h_/UP_2h_) values <1 or >1 correspond to gene downregulation or upregulation, respectively (dashed line). Relative gene expression values are reported in Supplementary Table S6. Values are expressed as mean ± SD of three independent replications with 20 seeds for each replication. Results of the Student’s *t*-test highlighting statistically significant differences are shown in Supplementary Table S7. Sm *Solanum melongena*, SOD superoxide dismutase, APX ascorbate peroxidase, OGG1 8-oxoguanine glycosylase/lyase, FPG formamidopyrimidine-DNA glycosylase, TDP1α tyrosyl-DNA phosphodiesterase 1α

In order to correlate ROS profiles with gene expression patterns, qRT-PCR analyses were carried on the different ‘67/3’ seed lots according to the experimental design (Fig. 2a) and results are shown in Fig. 5a. Values represent fold changes of transcript levels where FC = primed and dried-back seed (HPDB)/dry seed (DS). FC(HPDB/DS) values <1 or >1 correspond to gene downregulation or upregulation, respectively (Fig. 5a, dashed line). Relative gene expression values are reported in Supplementary Table S5. Apparently the HP72 treatment did not impact the expression of *SmSOD* and *SmAPX* genes in the high-quality seed lots. As for the low-quality samples, upregulation of *SmSOD* gene was detected in one lot (SL2, FC(HPDB/DS) = 3.44 ± 0.35) (Fig. 5a). In the case of DNA repair genes, the HP72 treatment resulted in *SmOGG1* upregulation only in SL1 and SL5, as shown by the FC(HPDB/DS) values (4.86 ± 0.20 and 4.17 ± 1.29, respectively) whereas no substantial changes were detected in SL2, SL3 and SL4. The *SmFPG* gene showed a seed lot-dependent expression profile in the high-quality samples and no significant changes occurred in the low-quality seeds. The effects of HP72 treatment on the *SmTDP1α* gene expression were heterogeneous across both the high-quality and low-quality seed lots (Fig. 5a). Results of the qRT-PCR analysis performed on imbibed seeds are shown in Fig. 5b. Values represent fold changes of transcript levels where FC = primed seed (HP_2h_)/unprimed seed (UP_2h_). FC(HP_2h_/UP_2h_) values <1 or >1 correspond to gene downregulation or upregulation, respectively (Fig. 5b, dashed line). Relative gene expression values are reported in Supplementary Table S6. At 2 h of imbibition hydropriming did not trigger substantial upregulation of the *SmSOD* gene in almost all the seed lots (Fig. 5b), however upregulation of *SmAPX* gene was evident in both high- and low-quality seed lots. The estimated FC(HP_2h_/UP_2h_) values were 9.23 ± 2.69 (SL1), 4.13 ± 1.18 (SL2), 1.61 ± 0.47 (SL3), 2.66 ± 1.59 (SL4), and 0.83 ± 0.16 (SL5). Upregulation of *SmOGG1* gene also occurred in almost all the tested seed lot except for SL1. As shown in Fig. 5b, the estimated FC(HP_2h_/UP_2h_) values were 2.13 ± 0.54 (SL2), 2.98 ± 0.66 (SL3), 1.55 ± 0.40 (SL4), and 4.46 ± 0.82 (SL5). Following imbibition, the *SmFPG* gene was downregulated in all the tested seed lots. The *SmTDP1α* gene expression showed fluctuations in all the tested seed lots (Fig. 5b).

## Discussion

In this work, we provide for the first time a molecular landscape of the pre-germinative metabolism in primed eggplant seeds, by integrating the expression patterns of antioxidant/DNA repair genes that play pivotal roles during imbibition with free radical profiles. The first aim of the investigation was the identification of hallmarks useful to monitor the seed response to priming and discriminate between high- and low-quality lots. The HP72 treatment hereby developed accelerates germination in the *S. melongena* inbred line ‘67/3’, resulting in beneficial effects, thus providing a useful technological solution. However, a better understanding of the molecular background will lead to significant advancements not only in eggplant but also in other valuable horticultural crops^4,7^.

The biochemical events triggered by water uptake during seed imbibition are associated with ROS accumulation, e.g. the respiratory burst within mitochondria generates the superoxide anion that is dismutated to hydrogen peroxide and the extracellular peroxidases that produce superoxide radicals then converted to hydrogen peroxide^38^. ROS are source of oxidative damage that is counteracted by the seed through the activation of the antioxidant defense and DNA repair, however these radical species also contribute to germination as specific oxidative modifications of storage proteins act as signals to trigger reserves mobilization^39^. The eggplant seeds revealed a boost in ROS levels at 24 h of hydropriming, followed by a progressive decrease throughout the treatment and the levels detected after dry-back were even lower compared to the dry seed. Accordingly, Kubala et al.^40^ found enhanced ROS levels in rape (*Brassica napus* L.) seeds subjected to osmopriming and a decrease during the subsequent drying. However, high ROS amounts were measured during the germination tests in both unprimed and primed *Brassica* seeds, differently from what observed in eggplant. As priming brings the seed to an advanced developmental stage^41^, it is possible that the ROS peak detected during hydropriming simply anticipates the physiological boost that takes place at 8 h of imbibition in the unprimed seeds.

Changes in free radicals during hydropriming, dry-back and subsequent germination are determined by the ROS-scavenging activity of antioxidant enzymes, among which ascorbate peroxidase and superoxide dismutase. Species-specific dynamics in ROS production and activity of scavenging enzymes during germination have been described, suggesting that the overall interplay between these two players can be tightly rearranged^38^. The upregulation of *SmAPX* gene provides evidence that eggplant seeds are dealing with the rise in ROS during hydropriming. On the other hand, it is possible that the downregulation of *SmSOD* gene reflects a crucial step in the pre-germinative metabolism, as highlighted by Oracz et al.^42^. These authors reported that both inhibition of SOD activity and increased activity of ROS-producing enzymes were able to promote ROS-mediated protein carbonylation, and trigger in sunflower (*Helianthus annuus* L.). A peak in *SmSOD* gene expression occurred during post-priming germination of ‘67/3’ seeds, between the 16 and 24 h timepoints (Fig. 3b) and this is in agreement with the documented increase in SOD activity of osmoprimed seeds from different crop plants^43,44^.

Maintenance of genome integrity is another big challenge for the seed^13^, and the controlled rehydration carried through priming promotes the cellular repair activities including the removal of DNA lesions^40,45^. As for eggplant, among the DNA repair genes hereby tested only *SmOGG1* showed upregulation during the HP72 treatment (Fig. 3a), suggesting that repair of oxidative DNA damage was requested particularly within the first 24 h of hydropriming. Later on, a peak in *SmOGG1* gene expression occurred in the primed seeds during the dry-back step, as evidenced by the comparison between the last timepoint of hydropriming (Fig. 3a, 72 h) and the starting point of the germination test (Fig. 3b, 0 h), supporting for an active response to genotoxic stress in agreement with Chen et al.^15^ who showed that overexpression of *AtOGG1* gene in *Arabidopsis* resulted in lower DNA damage accumulation during the late phase of seed maturation. More recently, Parreira et al.^46^ also demonstrated that activation of DNA repair at the onset of the desiccation phase is a molecular signature of seed maturation in common bean (*Phaseolus vulgaris* L.). When considering the response of imbibed seeds (Fig. 3b), hydropriming resulted in the early upregulation of *SmOGG1* gene (2 h) and *SmFPG* gene (4 and 16 h of imbibition), and there were no evident peaks in *SmTDP1α* gene expression.

A major challenge of seed priming is to design protocols that can work independently of the seed lot quality. Thus, the investigation was extended to eggplant seed lots showing variable germination profiles to test the reproducibility of the HP72 treatment. Furthermore, as the early upregulation of *SmOGG1* gene in primed seeds at 2 h of imbibition appeared as a promising hallmark, the subsequent investigation on eggplant was restricted within this timepoint, to verify whether the observed molecular landscape was reproducible in other seed lots. Indeed, shortcut protocols would be an ideal solution to speed-up the routine assessment of seed quality. Molecular tools as those investigated in the present work might be used to integrate other innovative approaches for assessing germination, e.g. NIR (Near Infra Red) spectroscopy. A striking difference between the high-quality and low-quality eggplant seed lots was that the primed, dried-back seeds with low germinability contained enhanced ROS levels, compared to the unprimed, and this profile was maintained also at 2 h of imbibition (Fig. 4b). This excessive ROS content might be detrimental and impair eggplant germination in the low-quality seed lots. At the level of dry seed, either in presence or absence of hydropriming, the *SmSOD* and *SmAPX* genes showed differences in the expression profiles (Fig. 5), resulting in lot-specific responses. The upregulation of *SmAPX* gene at 2 h of imbibition (Fig. 5b) was a common hallmark of high- and low-quality seed lots, symptomatic of an early antioxidant response triggered by priming. Despite this, the low-quality eggplant seeds were not able to restore an optimal germination performance (Table 2). Although lot-specific profiles were observed, the level of *SmOGG1* transcript was in most cases higher in the high-quality seeds, compared to the low quality ones (Fig. 5a, b). Overall, the reported data highlight the complexity of the molecular landscape depicted by the expression profiles of *SmOGG1*, *SmFPG*, and *SmTDP1α* genes in each specific seed lot. Looking at the cumulative effect of the gene expression profiles during hydropriming and subsequent germination as well as across lots, it seems that the repair machinery of the eggplant seed is endowed with a remarkable capacity to adapt to the changes induced by priming in the cell metabolism and physiology.

## Conclusion

This study explored for the first time the dynamics of the pre-germinative metabolism in eggplant, a valuable horticultural crop, investigating the impact of hydropriming, and looking for useful molecular hallmarks within a set of antioxidant and DNA repair genes that play a pivotal role during early seed imbibition. The investigation also faced the issues of seed quality by using lots with variable germinability. The plasticity of the seed pre-germinative metabolism, stimulated by priming, imposes a plethora of heterogeneous molecular responses, such as those disclosed in the present work, providing a valuable contribution to fill the current knowledge gap on the molecular bases of seed priming. On the other hand, the variability and heterogeneity hereby observed impair the search for molecular markers useful to speed-up diagnostics of seed quality. As a complex trait, the seed response to priming is difficult to dissect. The temporal sequence of molecular events influenced by environmental and genetic factors that come along with the treatment has to be elucidated. Even more difficult it will be to clarify how these events are coordinated to ensure seed quality. The information hereby gathered will help to establish future research work dealing with other basic aspects of the DNA damage response in horticultural crops, especially Solanaceae, and their wild relatives that face serious seed quality issues. As for the specific case of eggplants, a larger screening, at the level of seed lots and plant genotypes, will be necessary to accomplish the several open questions that still prevent priming to become a reproducible and reliable technique and support effective agricultural strategies.

## Materials and methods

### Seeds, germination tests, and priming

Fresh seeds of *S. melongena* L. (inbred line ‘67/3’ developed by Dr. Giuseppe Leonardo Rotino at CREA-GB, Montanaso Lombardo) were extracted from physiologically ripe fruits produced by plants cultivated in an open field at CREA-GB in Montanaso Lombardo (LO, Italy). For germination tests, seeds were transferred to Petri dishes (diameter 90 mm) containing two filter papers moistened with 2.5 ml of H_2_O, sealed and kept in a growth chamber at 24 °C under light conditions with photon flux density of 150 μmol m^−2^ s^−1^, photoperiod of 16/8 h and 70–80% relative humidity. Seeds with protrusion of the primary radicle were considered germinated and counted every day after imbibition. Germination parameters, calculated as described by Ranal and Garcia de Santana^37^ are listed in Supplementary Table S8. For priming, 45 seeds (15 seeds for each replicate) were soaked at 24 °C for the indicated time (24, 48, 72, and 96 h) in 400 ml H_2_O under aeration produced by a Wave Air Pump Mouse 2 Beta aerator (De Jong Marinelife B.V., The Netherlands) with the following parameters: 220–240 V, 50 Hz, 2.3 W, output 1.8 l min^−1^, pressure 0.012 MPa. For dehydration (Dry-Back, DB) primed seeds were transferred into glass tubes, placed between two cotton disks, covered with silica beads (disidry® Orange Silica Gel, The Aerodyne, Florence, Italy) with a seed: silica ratio of 1:10, and kept at 24–25 °C. Seed fresh weight was monitored every 15 min until the weight of dry seed was reached. Germination tests were carried out with unprimed (UP) and primed (HP) seeds as follows. For each treatment, three independent replications with 20 seeds per replication were analyzed. Seeds and seedlings were harvested at the indicated timepoints, the fresh weight was measured and samples were frozen using liquid N_2_ and stored at −80 °C for subsequent molecular analyses.

### ROS detection

ROS levels were quantified in dry and imbibed seeds, using the fluorogenic dye 2′,7′-dichlorofluorescein diacetate (DCFH-DA; Sigma-Aldrich, Milan Italy). The dye is converted to a non-fluorescent molecule following deacetylation mediated by cellular esterases, and it is subsequently oxidized by ROS into the fluorescent compound 2′,7′-dichlorofluorescein. DFC can be detected by fluorescence spectroscopy with maximum excitation and emission spectra of 495 nm and 529 nm, respectively. The assay was carried as described by Macovei et al.^47^, with the following modifications. *S. melongena* seeds were collected at the indicated timepoints and dried on filter paper. Samples (three seeds per timepoint) were incubated for 15 min with 50 μl of 10 μM DCFH-DA and subsequently fluorescence at 517 nm was determined using a Rotor-Gene 6000 PCR apparatus (Corbett Robotics, Brisbane, Australia), setting the program for one cycle of 30 s at 25 °C. As negative control, a sample containing only DCFH-DA was used to subtract the baseline fluorescence. Relative fluorescence was calculated by normalizing samples to controls and expressed as R.F.U.

### RNA extraction, cDNA synthesis, and quantitative real-time polymerase chain reaction

RNA isolation was carried out using the TRIZOL® Reagent (Fisher Molecular Biology, Trevose, U.S.A.) according to the supplier’s indications. cDNAs were obtained using the RevertAid First Strand cDNA Synthesis Kit (Thermo Fisher Scientific, Milan, Italy) according to the manufacturer’s suggestions. Quantitative real-time polymerase chain reaction (qRT-PCR) was performed with the Maxima SYBR Green qPCR Master Mix (2×) (Thermo Fisher Scientific) according to supplier’s indications, using a Rotor-Gene 6000 PCR apparatus (Corbett Robotics Pty Ltd). Amplification conditions were as follows: denaturation at 95 °C for 10 min, and 45 cycles of 95 °C for 15 s and 60 °C for 30 s and 72 °C for 30 s. Oligonucleotide primers were designed using the Real-Time PCR Primer Design program Primer3Plus (https://primer3plus.com) from GenScript and further validated through the online software Oligo Analyzer (https://eu.idtdna.com/calc/analyzer) (Supplementary Table S9). Quantification was carried out using *SmGADPH* (*glyceraldehyde 3-phosphate dehydrogenase*, Accession No. AB110609.1) and *SmAPRT* (*adenine phosphorybosyl transferase*, Accession No. JX448345.1)^48,49^ as reference genes for the experimental conditions (treated versus untreated) used in this work. Selection was performed using the GeNorm algorithm (https://genorm.cmgg.be) (Supplementary Information, Supplementary Fig. S2). The raw, background-subtracted fluorescence data provided by the Rotor-Gene 6000 Series Software 1.7 (Corbett Robotics) was used to estimate PCR efficiency (E) and threshold cycle number (*C*_t_) for each transcript quantification. The Pfaffl method^50^. was used for relative quantification of transcript accumulation and statistical analysis was performed with REST2009 Software V2.0.13 (Qiagen GmbH, Hilden, Germany). The following genes were tested: *SmOGG1* (*8-oxoguanine glycosylase/lyase*, SMEL_004g210790.1)*, SmFPG* (*formamidopyrimidine-DNA glycosylase*, SMEL_003g194660.1), *SmTDP1α* (*tyrosyl-DNA phosphodiesterase 1α*, SMEL_003g171200.1), *SmAPX* (*ascorbate peroxidase*, SMEL_006g245760.1.01), and *SmSOD* (*superoxide dismutase*, SMEL_001g139700.1.01).

### Statistical analysis

The effects of priming versus unprimed control in terms of germination percentage, days, and their interaction were analyzed using two-way ANOVA (analysis of variance) (‘*’*P* < 0.05; ‘**’*P* < 0.01, ‘***’*P* < 0.001, ‘****’*P* < 0.0001) carried out with the statistical software GraphPad Prism 8 (GraphPad Software Inc., California). Comparison between unprimed control and different priming treatments were carried out as follows. For each treatment, three biological replicates were considered. Means were compared using the post-hoc Tukey’s HSD (honest significant difference) test. Means with a significance value lower than 0.05 (*P* ≤ 0.05) were considered statistically different. Statistical analysis of phenotyping data and qRT-PCR data were performed using the Student’s *t*-test. Asterisks indicate statistically significant differences determined using Student’s *t*-test (‘*’*P* < 0.05; ‘**’*P* < 0.01, ‘***’*P* < 0.001). The efficacy of treatment versus unprimed control was carried out using Mann–Whitney test (‘*’*P* < 0.05; ‘**’*P* < 0.01, ‘***’*P* < 0.001).

## Supplementary information

Supplementary Material Revised

Supplementary Material Revised Marked-Up
