# Supplementary material for: Molecular dynamics of pre-germinative metabolism in primed eggplant (Solanum melongena L.) seeds
Source: Hortic Res. 2020 Jun 1;7:87. doi: 10.1038/s41438-020-0310-8 (PMC7261768; doi:10.1038/s41438-020-0310-8)
Supplement: Supplementary file 1 — Supplementary Material Revised [file 41438_2020_310_MOESM1_ESM.doc]

**Supplementary Information**

**Molecular dynamics of pre-germinative metabolism in primed eggplant *(Solanum melongena* L.) seeds**

Chiara Forti, Valentino Ottobrino, Laura Bassolino, Laura Toppino, Giuseppe Leonardo Rotino, Andrea Pagano, Anca Macovei, Alma Balestrazzi

**Impact of the HP72 protocol on the growth of *S. melongena* seedlings**

Seven-day old seedlings from unprimed (UP) and primed (HP72) seeds were evaluated in terms of fresh/dry weight, and radicle length. Results are shown in Supplementary Table S1. Statistical analysis highlighted a significant (*P* < 0.05) increase in fresh weight only in seedlings developed from HP72 seeds, compared to UP.

**Table S1 Results of phenotyping analyses performed on seven-day** **old *S. melongena* seedlings (line ‘67/3’) developed from seeds collected during year 2016 treated with hydropriming (HP72) and untreated (UP). Values are expressed as mean ± SD (standard deviation) of three independent replications with 15 seedlings for each replication (3 in total). Asterisks indicate statistically significant differences determined using Student’s *t*-test. (*) *P* < 0.05; (**) *P* < 0.01; (***) *P* < 0.001**

| **Parameter** | **UP** | **HP72** |
| --- | --- | --- |
| fresh weight (mg/ 15 seedling)  dry weight (mg/15 seedling)  radicle length (mm) | 37.40 ± 0.168  4.65 ± 0.029  38.50 ± 2.12 | 42.5 ± 0.028*  3.6 ± 0.005  47.0 ± 21.21 |

**Table S2** **Relative gene expression values measured in dry seeds (DS) of *S. melongena* (line ‘67/3’), seeds treated with hydropriming (HP) for 24, 48, and 72 h, and primed seeds subjected to dry-back (HPDB). Data are means ± SD (standard deviation) from three biological replicates. FPG, formamidopyrimidine DNA glycosylase. OGG1, 8-oxoguanine glycosylase/lyase. TDP, tyrosyl-DNA phosphodiesetrase. APX, ascorbate peroxidase. SOD, superoxide dismutase. *Sm*, *Solanum melongena***

| **Gene** | **DS** | **24h** | **48h** | **72h** | **HPDB** |
| --- | --- | --- | --- | --- | --- |
| *SmOGG1* | 0.623 ± 0.297 | 0.825 ± 0.230 | 0.400 ± 0.089 | 0.360 ± 0.083 | 2.079 ± 0.350* |
| *SmFPG* | 10.482 ± 2.243 | 2.671 ± 0.159* | 0.867 ± 0.178* | 0.906 ± 0.121* | 0.972 ± 0.085* |
| *SmTDP1α* | 2.442 ± 0.434 | 1.660 ± 0.007 | 1.005 ± 0.003* | 0.994 ± 0.000* | 0.020 ± 0.0003* |
| *SmAPX* | 0.145 ± 0.052 | 0.358 ± 0.124 | 0.185 ± 0.085 | 0.271 ± 0.163 | 0.238 ± 0.020 |
| *SmSOD* | 28.623 ± 4.842 | 3.560 ± 0.302* | 6.381 ± 0.979* | 2.097 ± 0.322* | 2.547 ± 0.818* |

**Table S3.** **Relative gene expression values measured during germination in *S. melongena* (line ‘67/3’) unprimed (UP) seeds (A) and primed (HP) seeds (B). Seeds treated with hydropriming for 72 h and subsequently dried-back. Data are means ± SD (standard deviation) from three biological replicates. FPG, formamidopyrimidine DNA glycosylase. OGG1, 8-oxoguanine glycosylase/lyase. TDP, tyrosyl-DNA phosphodiesetrase. APX, ascorbate peroxidase. SOD, superoxide dismutase. *Sm*, *Solanum melongena***

**(A)**

| **Gene** | **Unprimed (U)** | | | | | |  | | | |
| --- | --- | --- | --- | --- | --- | --- | --- | --- | --- | --- |
|  | **0h** | **2h** | **4h** | **8h** | **16h** | **24h** | | **48h** | **72h** | **RD** |
| *SmOGG1* | 0.623 ± 0.297 | 1.069 ± 0.035 | 0.727 ± 0.170 | 0.530 ± 0.076 | 1.700 ± 0.099 | 9.107 ± 2.38 | | 0.539 ± 0.026 | 0.352 ± 0.022 | 1.286 ± 0.080 |
| *SmFPG* | 10.482 ± 2.243 | 22.761 ± 5.604 | 3.395 ± 1.200 | 0.761 ± 0.105 | 0.905 ± 0.055 | 0.799 ± 0.114 | | 1.961 ± 0.180 | 1.049 ± 0.190 | 6.382 ± 1.147 |
| *SmTDP1α* | 2.442 ± 0.434 | 0.668 ± 0.309 | 0.420 ± 0.116 | 0.361 ± 0.033 | 2.602 ± 0.663 | 2.136 ± 0.49 | | 0.312 ± 0.008 | 0.395 ± 0.084 | n.d. |
| *SmAPX* | 0.145 ± 0.052 | 0.356 ± 0.031 | 1.315 ± 0.375 | 0.850 ± 0.171 | 0.538 ± 0.195 | 0.165 ± 0.017 | | 0.598 ± 0.061 | 0.087 ± 0.044 | 0.526 ± 0.123 |
| *SmSOD* | 28.623 ± 4.842 | 7.540 ± 1.707 | 10.320 ± 0.573 | 3.220 ± 0.700 | 1.267 ± 0.081 | 0.941 ± 0.043 | | 1.242 ± 0.268 | 1.175 ± 0.181 | 9.862 ± 1.005 |

(B)

| **Gene** | **Primed (HP)** | | | | | |  | | | |
| --- | --- | --- | --- | --- | --- | --- | --- | --- | --- | --- |
|  | **0h** | **2h** | **4h** | **8h** | **16h** | **24h** | | **48h** | **72h** | **RD** |
| *SmOGG1* | 2.079 ± 0.350* | 4.790 ± 1.032* | 1.512 ± 0.156* | 0.446 ± 0.145 | 0.074 ± 0.004 | 0.181 ± 0.019 | | 0.350 ± 0.216 | 0.281 ± 0.070 | 0.042 ± 0.021* |
| *SmFPG* | 0.972 ± 0.085* | 3.533 ± 0.756* | 8.252 ± 2.033* | 0.660 ± 0.478 | 1.338 ± 0.320 | 1.161 ± 0.080* | | 0.923 ± 0.229* | 1.029 ± 0.136 | 0.851 ± 0.018* |
| *SmTDP1α* | 0.020 ± 0.0003* | 0.007 ± 0.002 | 0.589 ± 0.045 | 0.171 ± 0.032* | 0.505 ± 0.373 | 0.217 ± 0.017* | | 0.222 ± 0.069 | 0.364 ± 0.116 | n.d. |
| *SmAPX* | 0.238 ± 0.020 | 0.443 ± 0.068 | 0.359 ± 0.054* | 0.474 ± 0.173 | 1.840 ± 0.456 | 0.312 ± 0.027* | | 0.144 ± 0.070* | 0.121 ± 0.016 | 0.607 ± 0.600 |
| *SmSOD* | 2.547 ± 0.818* | 12.948 ± 6.77 | 15.080 ± 4.464 | 1.596 ± 0.046 | 64.675 ± 7.702* | 0.629 ± 0.027* | | 0.342 ± 0.007* | 1.238 ± 0.154 | 16.937 ± 1.441* |

**Table S4. Results of the Student’s *t* test highlighting statistically significant differences between *S. melongena* (line ‘67/3’) seeds collected during the HP72 treatment (at 24 h, 48 h, and 72 h) and compared to the dry seed and during germination test. HP, primed seeds. DS, dry seed. UP, unprimed seeds.. FPG, formamidopyrimidine DNA glycosylase. OGG1, 8-oxoguanine glycosylase/lyase. TDP, tyrosyl-DNA phosphodiesetrase. APX, ascorbate peroxidase. SOD, superoxide dismutase. *Sm*, *Solanum melongena***

| **dHP**  Comparison | ***SmOGG1***  *P* | ***SmSOD***  *P* | ***SmAPX***  *P* | ***SmTdp1α***  *P* | ***SmFPG***  *P* |
| --- | --- | --- | --- | --- | --- |
|
| 24h-48h | 0.07 | 0.03 * | 0.13 | 0.000001 *** | 0.0002 *** |
| 24h-72h | 0.06 | 0.005 ** | 0.51 | 0.00004 *** | 0.0002 *** |
| 48h-72h | 0.60 | 0.01 ** | 0.48 | 0.03 * | 0.77 |
| **HPDB/UP**  Comparison | ***SmOGG1***  *P* | ***SmSOD***  *P* | ***SmAPX***  *P* | ***SmTdp1α***  *P* | ***SmFPG***  *P* |
|
| UP-2h | 0.88 | 0.03 * | 0.12 | 0.54 | 0.15 |
| UP-4h | 0.35 | 0.03 * | 0.06 | 0.04 * | 0.04 * |
| UP-8h | 0.18 | 0.01 ** | 0.07 | 0.002 ** | 0.17 |
| UP-16h | 0.13 | 0.004 ** | 0.23 | 0.06 | 0.03 * |
| UP-24h | 0.80 | 0.0001 *** | 0.80 | 0.005 ** | 0.002 ** |
| UP-48h | 0.17 | 0.01 ** | 0.05 * | 0.03 * | 0.04 * |
| UP-72h | 0.18 | 0.02 * | 0.79 | 0.09 | 0.04 * |
| UP-RD | 0.13 | 0.004 ** | 0.29 | n.d. | 0.13 |
| 2h-4h | 0.02 * | 0.65 | 0.02 * | 0.04 * | 0.04 * |
| 2h-8h | 0.01 ** | 0.05 * | 0.29 | 0.003 ** | 0.19 |
| 2h-16h | 0.01 ** | 0.004 ** | 0.13 | 0.06 | 0.03 * |
| 2h-24h | 0.01 ** | 0.07 | 0.02 * | 0.007 ** | 0.0009 *** |
| 2h-48h | 0.006 ** | 0.04 * | 0.009 ** | 0.03 * | 0.05 * |
| 2h-72h | 0.01 ** | 0.16 | 0.19 | 0.09 | 0.04 * |
| 2h-RD | 0.01 ** | 0.82 | 0.70 | n.d. | 0.49 |
| 4h-8h | 0.05 * | 0.06 | 0.19 | 0.07 | 0.06 |
| 4h-16h | 0.02 * | 0.004 ** | 0.10 | 0.05 * | 0.14 |
| 4h-24h | 0.02 * | 0.09 | 0.01 ** | 0.04 * | 0.14 |
| 4h-48h | 0.70 | 0.04 * | 0.72 | 0.10 | 0.04 * |
| 4h-72h | 0.05 * | 0.27 | 0.08 | 0.31 | 0.07 |
| 4h-RD | 0.02 * | 0.43 | 0.25 | n.d. | 0.04 * |
| 8h-16h | 0.01 ** | 0.004 ** | 0.11 | 0.02 * | 0.28 |
| 8h-24h | 0.01 ** | 0.12 | 0.009 ** | 0.002 ** | 0.29 |
| 8h-48h | 0.52 | 0.053 | 0.16 | 0.19 | 0.38 |
| 8h-72h | 0.81 | 0.04 * | 0.12 | 0.23 | 0.81 |
| 8h-RD | 0.01 ** | 0.003 ** | 0.45 | n.d. | 0.18 |
| 16h-24h | 0.08 | 0.004 ** | 0.25 | 0.16 | 0.93 |
| 16h-48h | 0.12 | 0.004 ** | 0.10 | 0.04 * | 0.03 * |
| 16h-72h | 0.01 ** | 0.004 ** | 0.20 | 0.13 | 0.19 |
| 16h-RD | 0.35 | 0.004 ** | 0.13 | n.d. | 0.03 * |
| 24h-48h | 0.11 | 0.0008 *** | 0.009 ** | 0.04 * | 0.0009 *** |
| 24h-72h | 0.01 ** | 0.1 | 0.60 | 0.10 | 0.12 |
| 24h-RD | 0.46 | 0.01 ** | 0.21 | n.d. | 0.002 *** |
| 48h-72h | 0.59 | 0.03 * | 0.08 | 0.47 | 0.08 |
| 48h-RD | 0.11 | 0.005 ** | 0.24 | n.d. | 0.051 |
| 72h-RD | 0.01 ** | 0.03 * | 0.42 | n.d. | 0.04 * |

**Table S5. Relative gene expression values measured in *S. melongena* (line ‘67/3’) seed lots collected at different years and subjected to HP treatment. UP, unprimed dry seeds. HPDB, seeds treated with hydropriming for 72 h and subsiquently dried-back. Data are means ± SD (standard deviation) from three biological replicates. SL, seed lot. FPG, formamidopyrimidine DNA glycosylase. OGG1, 8-oxoguanine glycosylase/lyase. TDP, tyrosyl-DNA phosphodiesetrase. APX, ascorbate peroxidase. SOD, superoxide dismutase. *Sm*, *Solanum melongena***

| **Gene** | **SL1 (2014)** | | **SL2 (2015)** | | **SL3 (2016)** | | **SL4 (2017)** | | **SL5 (2018)** | |
| --- | --- | --- | --- | --- | --- | --- | --- | --- | --- | --- |
|  | UP | HPDB | UP | HPDB | UP | HPDB | UP | HPDB | UP | HPDB |
| *SmOGG1* | 0.86 ± 0.13 | 4.180 ± 0.31* | 0.62 ± 0.02 | 0.044 ± 0.03 | 1.01 ± 0.17 | 1.39 ± 0.21 | 1.05 ± 0.07 | 1.11 ± 0.06 | 0.62 ± 0.3 | 2.08 ± 0.35* |
| *SmTDP1α* | 0.39 ± 0.01 | 0.56 ± 0.22 | 0.88 ± 0.73 | 1.91 ± 1.53 | 0.1 ± 0.02 | 0.43 ± 0.01 | 0.25 ± 0.008 | 0.32 ± 0.16 | 2.44 ± 0.43 | 0.02 ± 0.01* |
| *SmAPX* | 3.20 ± 1.22 | 1.650 ± 0.61 | 0.60 ± 0.07 | 0.04 ± 0.01* | 1.10 ± 0.24 | 1.35 ± 0.22 | 1.54 ± 0.55 | 1.23 ± 0.66 | 0.15 ± 0.05 | 0.24 ± 0.02 |
| *SmSOD* | 5.94 ± 0.61 | 0.007 ± 0.002* | 0.62 ± 0.08 | 2.13 ± 0.16* | 11.73 ± 3.81 | 2.10 ±1.61* | 9.85 ± 3.44 | 0.99 ± 0.67 | 28.62 ± 4.84 | 2.55 ± 0.82* |

**Table S6. Relative gene expression values measured in *S. melongena* (line ‘67/3’) seed lots collected at different years and subjected to HP treatment. UP2, unprimed seeds collected at 2 h of imbibition. HPDB2, primed seeds collected at 2 h of imbibition. Data are means ± SD (standard deviation) from three biological replicates. SL, seed lot. FPG, formamidopyrimidine DNA glycosylase. OGG1, 8-oxoguanine glycosylase/lyase. TDP, tyrosyl-DNA phosphodiesetrase. APX, ascorbate peroxidase. SOD, superoxide dismutase. *Sm*, *Solanum melongena***

| **Gene** | **SL1 (2014)** | | **SL2 (2015)** | | **SL3 (2016)** | | **SL4 (2017)** | | **SL5 (2018)** | |
| --- | --- | --- | --- | --- | --- | --- | --- | --- | --- | --- |
|  | UP2 | HPDB2 | UP2 | HPDB2 | UP2 | HPDB2 | UP2 | HPDB2 | UP2 | HPDB2 |
| *SmOGG1* | 0.60 ± 0.05 | 0.44 ± 0.19 | 0.24 ± 0.06 | 0.48 ± 0.03* | 0.44 ± 0.05 | 1.33 ± 0.13* | 0.78 ± 0.24 | 1.15 ± 0.15 | 1.07 ± 0.04 | 4.79 ± 1.03* |
| *SmTDP1α* | 0.25 ± 0.16 | 0.09 ± 0.03* | 0.14 ± 0.06 | 0.16 ± 0.01 | 0.51 ± 0.03 | 0.54 ± 0.02 | 0.12 ± 0.03 | 0.12 ± 0.06 | 0.69 ± 0.31 | 0.007 ± 0.002* |
| *SmAPX* | 0.19 ± 0.08 | 1.65 ± 0.79 | 0.40 ± 0.05 | 1.63 ± 0.47* | 0.79 ± 0.22 | 1.20 ± 0.13 | 0.62 ± 0.11 | 1.55 ± 0.35 | 0.36 ± 0.03 | 0.44 ± 0.07 |
| *SmSOD* | 1.19 ± 0.48 | 0.004 ± 0.0002 | 1.69 ± 0.09 | 1.20 ± 0.22* | 7.13 ± 2.49 | 1.18 ± 0.24 | 12.36 ± 1.82 | 9.01 ± 2.43 | 7.54 ± 1.71 | 12.95 ± 6.77 |

**Table S7. Results of the Student’s *t* test highlighting statistically significant differences between *S. melongena* (line ‘67/3’) seed lots collected at different years and subjected to HP treatment. HPDB, primed seeds. DS, dry seed. UP2, unprimed seeds collected at 2 h of imbibition. HPDB2, primed seeds collected at 2 h of imbibition. SL, seed lot. FPG, formamidopyrimidine DNA glycosylase. OGG1, 8-oxoguanine glycosylase/lyase. TDP, tyrosyl-DNA phosphodiesetrase. APX, ascorbate peroxidase. SOD, superoxide dismutase. *Sm*, *Solanum melongena***

| **HPDB/DS**  Comparison | ***SmOGG1***  *P* | ***SmSOD***  *P* | ***SmAPX***  *P* | ***SmTdp1α***  *P* | ***SmFPG***  *P* |
| --- | --- | --- | --- | --- | --- |
|
| SL1-SL2 | 0.00005 *** | 0.003 ** | 0.28 | 0.1 | 0.08 |
| SL1-SL3 | 0.0005 *** | 0.25 | 0.56 | 0.03 * | 0.11 |
| SL1-SL4 | 0.00004 *** | 0.31 | 0.85 | 0.77 | 0.07 |
| SL1-SL5 | 0.72 | 0.07 | 0.25 | 0.04 * | 0.06 |
| SL2-SL3 | 0.29 | 0.0004 *** | 0.04 * | 0.08 | 0.19 |
| SL2-SL4 | 0.67 | 0.0003 *** | 0.23 | 0.11 | 0.20 |
| SL2-SL5 | 0.21 | 0.003 ** | 0.04 * | 0.005 ** | 0.13 |
| SL3-SL4 | 0.16 | 0.89 | 0.72 | 0.03 * | 0.0001 *** |
| SL3-SL5 | 0.24 | 0.44 | 0.32 | 0.03 * | 0.00005 *** |
| SL4-SL5 | 0.20 | 0.54 | 0.33 | 0.08 | 0.06 |

| **HPDB2/UP2**  Comparison | ***SmOGG1***  *P* | ***SmSOD***  *P* | ***SmAPX***  *P* | ***SmTdp1α***  *P* | ***SmFPG***  *P* |
| --- | --- | --- | --- | --- | --- |
|
| SL1-SL2 | 0.03 * | 0.006 ** | 0.26 | 0.26 | 0.07 |
| SL1-SL3 | 0.02 * | 0.03 * | 0.15 | 0.37 | 0.08 |
| SL1-SL4 | 0.05 * | 0.007 ** | 0.18 | 0.48 | 0.06 |
| SL1-SL5 | 0.008 ** | 0.03 * | 0.13 | 0.25 | 0.06 |
| SL2-SL3 | 0.16 | 0.005 ** | 0.05 * | 0.55 | 0.08 |
| SL2-SL4 | 0.21 | 0.89 | 0.27 | 0.50 | 0.0008 *** |
| SL2-SL5 | 0.02 * | 0.07 | 0.04 * | 0.08 | 0.15 |
| SL3-SL4 | 0.04 * | 0.006 ** | 0.37 | 0.75 | 0.02 * |
| SL3-SL5 | 0.07 | 0.03 * | 0.09 | 0.08 | 0.02 * |
| SL4-SL5 | 0.01 ** | 0.07 | 0.18 | 0.06 | 0.37 |

**Table S8. Germination parameters used in this study to assess the efficacy of hydropriming on *S. melongena* L. seeds (inbred line ‘67/3’) as reported by Ranal and Garcia de Santana (2006). For each parameter, definition, formula, limits of measurement and unit are shown**

| **Parameter** | **Formula** | **Limits** | **Unit** |
| --- | --- | --- | --- |
| *G*  mean number of germinated seeds per day expressed in percentage | *G* = (100* n. of germinated seeds)/  Total n. of seeds | 0 ≤ *G* ≤ 100 | % |
| *MGT*  mean germination time (*) | 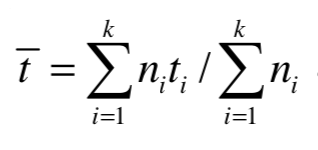 | 0 < *t* ≤ *k* | day |
| *CVG*  coefficient of velocity of germination | 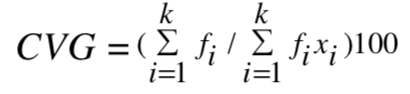 | 0 < *CVG* ≤ 100 | % |
| *MGR*  mean germination rate | *v* = *CV/100* | 0 < *v* ≤ 1 | day^-1^ |
| *U*  uncertainty associated to the distribution of the relative frequency of germination (**) | 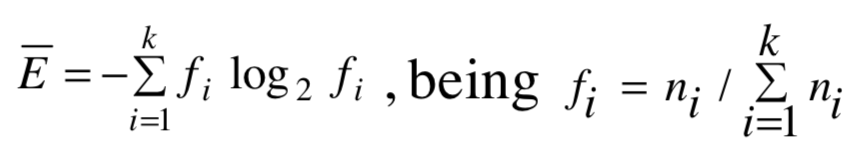 | 0 ≤ *U* ≤ log_2_ *n* | bit |
| *Z*  synchronization index (***) | 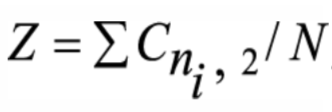 | 0 ≤ *Z* ≤ 1 | Unit less |

(*) *t_i_* is time from the start of the experiment to the *i^th^* observation (day); *n_i_*: number of seeds germinated in the time *i* (not the accumulated number, but the number correspondent to the *i^th^* observation), and *k* is the last time of germination.

(**) *f_i_* is the relative frequency of germination, *n_i_* the number of seeds germinated on the day *i*, and *k* the last day of observation

(***) *C_ni_, _2_* : combination of the seeds germinated in the time *i*, two together, and *n_i_* the number of seeds germinated in the time *i.*

**Reference**

Ranal MA, Garcia de Santana D. How and why to measure the germination process? *Brazilian J Bot.* 2006, **29**: 1-11.

**Table S9.** **List of oligonucleotide primers used for *q*RT-PCR analyses. For each oligonucleotide set, PCR efficiency is reported. FPG, formamidopyrimidine DNA glycosylase. OGG1, 8-oxoguanine glycosylase/lyase. TDP, tyrosyl-DNA phosphodiesetrase. APX, ascorbate peroxidase. SOD, superoxide dismutase. GAPDH, glyceraldehyde 3-phosphate dehydrogenase. PP2A, phosphatase 2A. TUB, tubulin. EF, elongation factor. APRT, adenine phosphorybosyl transferase**

| **Gene**  **(accession number)** | **Forward Primer (5’-3’)** | **Reverse Primer (5’-3’)** | **Efficiency** |
| --- | --- | --- | --- |
| *SmAPX*  (SMEL_006g245760.1.0) | GCAGTTTCCCATCCTCTCCC | GGTGGTTCTGGCTTGTCCTC | 1.73 |
| *SmSOD*  (SMEL_001g139700.1.0) | CTGGAAATGCTGGCGGAAG | GGAGGAATCAACCCTGGAGC | 1.81 |
| *SmOGG1*  (SMEL_004g210790.1) | TTATTGACCAGCAGCCCACA | ATACACCAGCAACACCCCTT | 1.70 |
| *SmFPG*  (SMEL_003g194660.1) | CCAAAAGAATACGGGAGGTGA | TTCTGGCTCTTCATCTTGACC | 1.73 |
| *SmTDP1α*    (SMEL_003g171200.1) | GCTTCACCAGGCAACAAACA | CCCACGCACTCTCATCAATC | 1.70 |
| *SmGAPDH*  (AB110609.1) | GGTGCCAAGAAGGTTGTGAT | GGTTACGATCAACGTGTTGC | 1.81 |
| *SmPP2Acs*  (AY325817.1) | GGACTCTCACCATCCCTTGA | ACCAACCCTTATAGTGGAG | 1.80 |
| *SmTUB*  (DQ205342.1) | CCAGACAGGATGATGCTCAC | TACCAGGAACTGTTGCTTCG | 1.80 |
| *SmEF1*  (X14449.1) | ACCAAGATTGACAGGCGTTC | GGAAACGACTTATGGGAGGT | 1.79 |
| *SmAPRT*  (X448345.1) | TGGCGCCTCATGATCCGATTCTTA | ACTCCAACACGCTCAAGAAGCCTA | 1.75 |

**
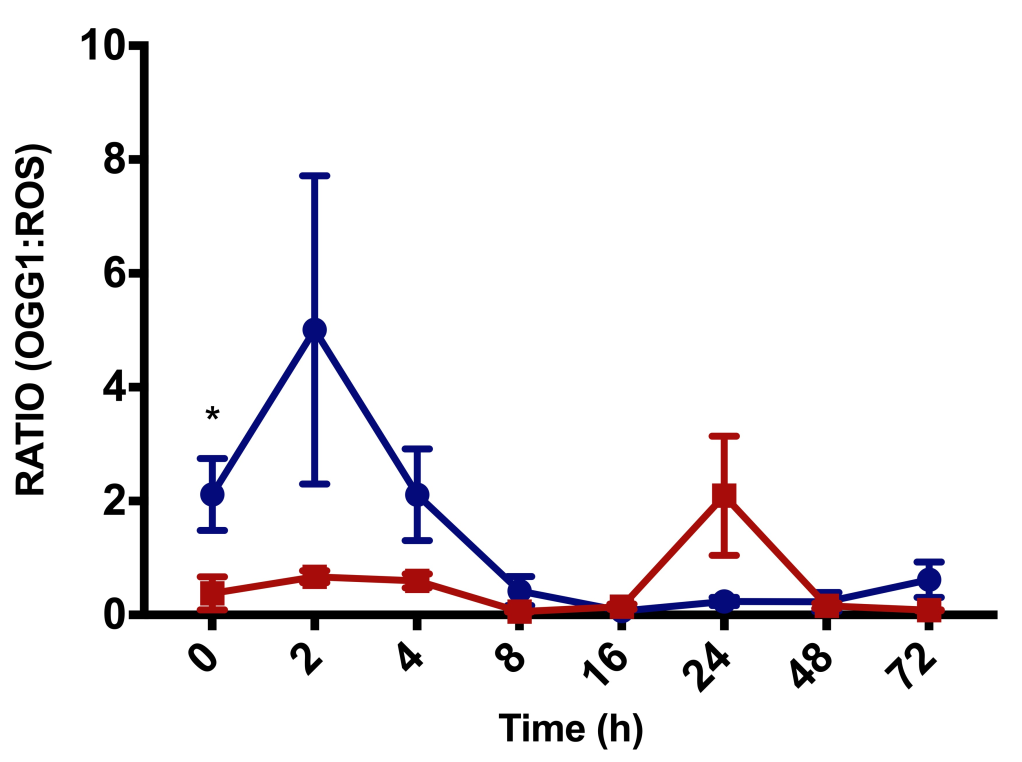
**

**Fig. S1** Ratio between the *SmOGG1* transcript level and the ROS amount (OGG1:ROS) calculated during the germination test (0 h, 2 h, 4 h, 8 h, 16 h, 24 h, 48 h, and 72 h) of eggplant seeds (*S. melongena* ‘67/3’ inbred line). Values are expressed as mean ± SD of three independent replications with 20 seeds for each replication. Asterisks indicate statistically significant differences determined using Student’s *t*-test (*P* < 0.05) (See Supplementary Table S10). OGG1, 8-oxoguanine glycosylase/lyase. ROS, reactive oxygen species

**Table S10. Results of the Student’s *t* test highlighting statistically significant differences between the *SmOGG1* transcript level and the ROS amount (OGG1:ROS) calculated during the germination test (0 h, 2 h, 4 h, 8 h, 16 h, 24 h, 48 h, and 72 h) of eggplant seeds (*S. melongena* ‘67/3’ inbred line).**

| **OGG1:ROS**  Comparison | *P* |
| --- | --- |
|
| UP:HPDB | 0.025 * |
| UP2:HPDB2 | 0.11 |
| UP4:HPDB4 | 0.08 |
| UP8:HPDB8 | 0.13 |
| UP16:HPDB16 | 0.07 |
| UP24:HPDB24 | 0.09 |
| UP48:HPDB48 | 0.55 |
| UP72:HPDB72 | 0.10 |

## Selection of reference genes for qRT-PCR analysis in S. melongena inbred line ‘67/3’

Reference genes were selected through GeNorm algorithm (https://genorm.cmgg.be) (Vandesompele et al., 2002) as follows. Five reference genes (*SmEF*, *SmPp2AC*s, *SmTUB*, *SmGAPDH* and *SmAPRT*) were tested, encoding proteins involved in basic cellular processes (housekeeping genes), commonly used as controls for gene expression analyses in plants since they are supposed to have a uniform expression profiles independent of spatial and temporal parameters and stress agents. Transcript levels were measured by *q*RT-PCR, using three independent cDNA samples prepared from: HP72-treated seeds (collected at 24, 48 and 72 h during hydropriming treatment and after dry-back), UP and HP72-treated seeds collected during germination tests (2 h, 4 h, 8 h, 16 h, 24 h, 48 h, 72 h and radicle protrusion stage-RD). A low value of the average expression stability, as calculated by GeNorm software, is indicative for a stable expression throughout the stress treatments. As a first step, each candidate gene was tested by *q*RT-PCR using as template cDNA samples representative of each timepoint of the experimental design. The constitutive transcriptional profile of the candidate reference genes was evaluated using the GeNorm tool (Fig. S2). Based on the M values provided by the software (Fig. S1), *SmGAPDH* and *SmAPRT* genes resulted as the most suitable as reference genes (*M* value of 1.66 and 1.58, respectively). The remaining genes (*SmEF, SmPp2ACs and SmTUB*) showed higher *M* values (values of 2.46, 2.10 and 1.77 respectively) and for this reason they were discarded.

**Reference**

Vandesompele J, De Preter K, Pattyn F, Poppe B, Van Roy N, De Paepe A, Speleman F. Accurate normalization of real-time quantitative RT-PCR data by geometric averaging of multiple internal control genes. *Genome Biol.* 2002, **3**: research0034.1.

**
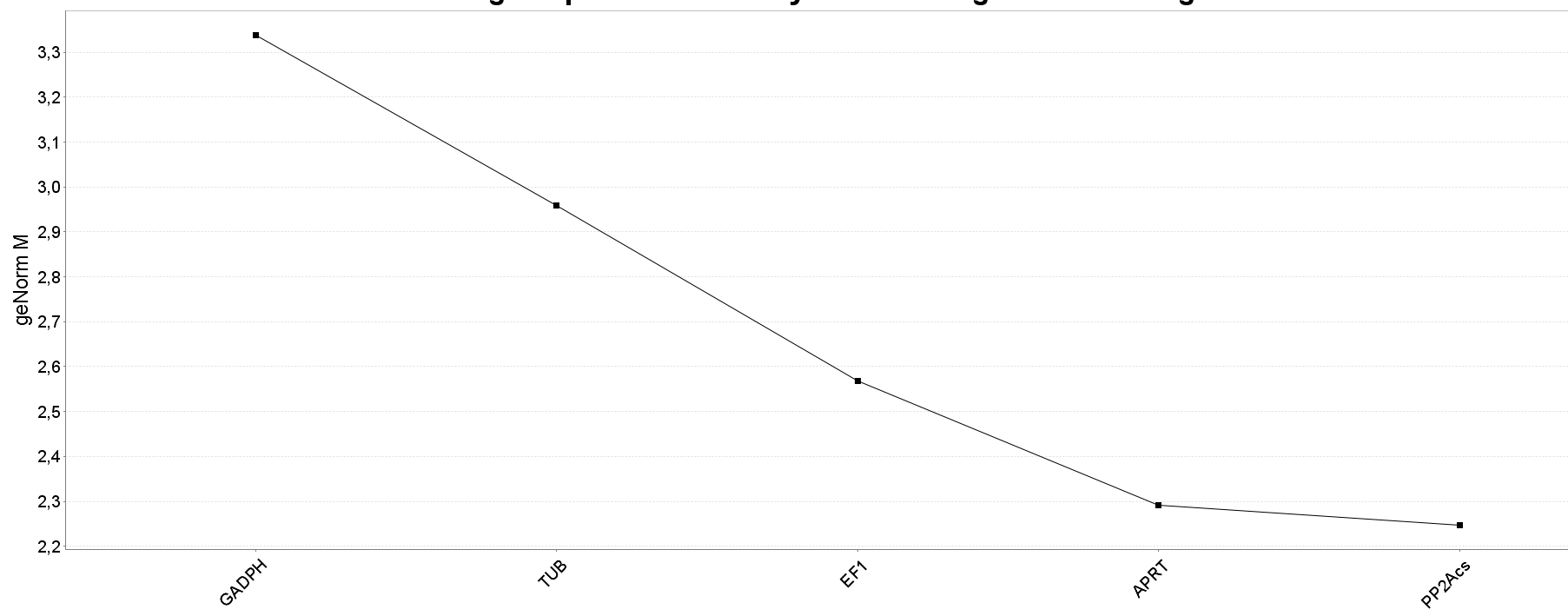
**

## Fig. S2 Selection of reference genes for qRT-PCR analysis in S. melongena inbred line ‘67/3’. Average expression stability values (M), calculated using GeNorm algorithm (https://genorm.cmgg.be), of the five putative reference genes analysed in primed and unprimed seeds throughout the tested time points of the experimental design. GAPDH, glyceraldehyde 3-phosphate dehydrogenase. PP2A, phosphatase 2A. TUB, tubulin. EF, elongation factor. APRT, adenine phosphorybosyl transferase

**Table S11. Results of statistical analysis carried out using Two-way ANOVA (F = 45.66; DF = 4; *P* = 0.0004***). Comparison between UP and HP24, HP48, HP72, HP96 were carried out using the Post-Hoc Tukey’s HSD test (*P* ≤ 0.05)**

| **UP vs HP** | **q** | **DF** | ***P*** | **Significance** |
| --- | --- | --- | --- | --- |
| 24 h | 4.090 | 61.70 | 0.0407 | * |
| 48 h | 4.674 | 61.39 | 0.0133 | * |
| 72 h | 6.141 | 62.00 | 0.0005 | *** |
| 96 h | 6.007 | 60.94 | 0.0007 | *** |
